# Rapid humoral immune responses are required for recovery from haemorrhagic fever with renal syndrome patients

**DOI:** 10.1080/22221751.2020.1830717

**Published:** 2020-10-21

**Authors:** Yaoni Li, Chuansong Quan, Weijia Xing, Peihan Wang, Jiming Gao, Zhenjie Zhang, Xiaolin Jiang, Chuanmin Ma, Michael J. Carr, Qian He, Lei Gao, Yuhai Bi, Hua Tang, Weifeng Shi

**Affiliations:** a Baoji Center Hospital, Baoji, People’s Republic of China; b Key Laboratory of Etiology and Epidemiology of Emerging Infectious Diseases in Universities of Shandong, Shandong First Medical University & Shandong Academy of Medical Sciences, Taian, People’s Republic of China; c School of Public Health, Shandong First Medical University & Shandong Academy of Medical Sciences, Taian, People’s Republic of China; d Institute of Immunology, Shandong First Medical University& Shandong Academy of Medical Sciences, Taian, People’s Republic of China; e Shandong Center for Disease Control and Prevention, Jinan, People’s Republic of China; f National Virus Reference Laboratory, School of Medicine, University College Dublin, Dublin, Ireland; g Global Institution for Collaborative Research and Education (GI-CoRE), Hokkaido University, Kita-ku, Japan; h School of Life Sciences, Shandong First Medical University & Shandong Academy of Medical Sciences, Taian, People’s Republic of China; i Key Laboratory of Pathogenic Microbiology and Immunology, Collaborative Innovation Center for Diagnosis and Treatment of Infectious Disease, Institute of Microbiology, Center for Influenza Research and Early Warning, Chinese Academy of Sciences, Beijing, People’s Republic of China

**Keywords:** Haemorrhagic fever with renal syndrome, Hantaan virus, humoral response, B cell, virus infection

## Abstract

Haemorrhagic fever with renal syndrome (HFRS) following Hantaan virus (HTNV) infection displays variable clinical signs. Humoral responses elicited during HTNV infections are considered important, however, this process remains poorly understood. Herein, we have investigated the phenotype, temporal dynamics, and characteristics of B-cell receptor (BCR) repertoire in an HFRS cohort. The serological proﬁles were characterized by a lowered expression level of nucleoprotein (NP)-speciﬁc antibody in severe cases. Importantly, B-cell subsets were activated and proliferated within the first two weeks of symptom onset and moderate cases reacted more rapidly. BCR analysis in the recovery phase revealed a dramatic increase in the immunoglobulin gene diversity which was more significantly progressed in moderate infections. In severe cases, B-cell-related transcription was lower with inflammatory sets overactivated. Taken together, these data suggest the clinical signs and disease recovery in HFRS patients were positively impacted by rapid and efficacious humoral responses.

## Introduction

Haemorrhagic fever with renal syndrome (HFRS) is caused by Hantaan virus (HTNV), a high-consequence hantavirus pathogen in the order *Bunyavirales* [1]. In addition to the well-recognized zoonotic transmission mode of contact with rodent reservoirs, or their excreta, the bite of parasitic mites is another route for transmission and maintenance of the agent. Trombiculid and gamasid mites have been identified as potential vectors and reservoir hosts for HTNV [2,3]. The clinical course of HFRS infection is highly variable, with infections ranging from asymptomatic seroconversion events to a febrile presentation with anuria necessitating dialysis, and even fatalities. Although effective prophylactic vaccines are available, >12,495 cases of HFRS were reported in mainland China in 2018, with a 0.8% case fatality rate (http://www.nhc.gov.cn/jkj/s2907/new_list.shtml?tdsourcetag=s_pcqq_aiomsg). The mechanisms of HTNV pathogenesis remain poorly understood. The intrinsic virulence of different HTNV strains is variable and host factors are also thought to exert a more important influence upon viral clearance and recovery following acute infection [4].

Previous studies have reported cytokine overexpression by macrophages, monocytes and lymphocytes, including interleukin (IL)-6, IL-8, tumour necrosis factor-α (TNF-α), interferon-β (IFN-β), and interferon-gamma-inducible protein (IP)-10, and this hypercytokinemia correlated with HFRS disease severity [5,6]. CD4^+^ T cells with broad antigenic repertoires possessing polyfunctional properties lead to less severe clinical outcomes, which may enhance the antiviral status of host cells and the cytotoxic effect of ThGranzyme B^+^ cells [7]. The CD8^low^ CD100^−^ subset is also activated and subsequently expresses more cytolytic effector molecules to combat HTNV infections [7]. It has also been noted that individuals harbouring human leukocyte antigen (HLA) alleles with HLA types B8, DRB1*0301 and DRB1*1101–1105 usually have higher viral loads, increasing the risk of developing severe disease [8–10]. Although virus-specific IgM is detectable simultaneously with the occurrence of clinical symptoms, and IgG of various subtypes is subsequently developed against viral structural proteins, the systematic mechanisms of virus-specific humoral responses to HTNV infection and their roles in the recovery are incompletely understood [11,12].

Exploring the host factors underlying the clinical spectrum of HTNV infection is essential for the timely implementation of effective therapeutic measures. In the present study, longitudinal blood samples were collected, including sera, plasma, and peripheral blood mononuclear cells (PBMCs), from laboratory-confirmed HTNV infections enrolled in Baoji city, from Shaanxi province in China for analysis of humoral responses. The profile of clinical features, including viral loads, systemic cytokine concentrations, and humoral responses, was comprehensively characterized. The primary objective was to understand efficacious humoral responses against HTNV infection resulting in viral clearance and disease resolution.

## Materials and methods

### Ethical approval

This study was approved by the ethics committee of the Shandong First Medical University & Shandong Academy of Medical Sciences. The study was followed by the principles of the Declaration of Helsinki, and the standards of Good Clinical Practice as defined by the International Conference on Harmonization (https://www.ich.org). A written informed consent was obtained from each HFRS patient or their guardians. The research-related information was used anonymously.

### Patients

Clinical specimens were collected from HFRS patients at Baoji central hospital from November 2018 to January 2019. NP-IgM and NP-IgG in serum specimens were detected by serological testing for HTNV diagnosis. According to the diagnostic criteria from the prevention and treatment strategy of HFRS promulgated by the Ministry of Health, People's Republic of China, the patients were classified into four clinical types: mild, moderate, severe, and critical [7]. To depict the dynamic changes of humoral response, the consecutive blood samples of HTNV patients with difference clinical types were collected.

### Clinical samples

The clinical outcomes of patients, obtained from routine blood test and urinalysis, were regularly recorded, as well as collection of fresh peripheral blood samples at different time points during hospitalization. A whole blood sample collected in a vacutainer (Becton Dickinson, New Jersey, USA) was used to separate the plasma stored at −80°C and PBMCs isolated by the method of Dakewe Biotech (Cat.DKW-KLST-015) were preserved in liquid nitrogen. Isolated sera were stored at −80°C. All controls were unexposed to any known potential risk factors, showed no symptoms of any infectious diseases, and had no known recent vaccination or condition or treatment that would interfere with immune responses [13].

### Viral RNA determination

The viral load in patients’ plasma was tested on an ABI 7500 Real-Time PCR system (Life Tech, USA). The oligonucleotide primers and probes were modified from those previously described [14]: forward primer: 5'-TRCAGAGGGAAATCAATGCC-3’, reverse primer: 5'-TGTTYARCTCATCTGGATCCTT-3’, probe: 5'-FAM-GATAGCCAGGCAGAAGGTGAGGGAT-TAMRA-3’. The S gene segment was cloned into the PET-20 vector and mRNA was synthesized following *in vitro* transcription using the Promega Kit (Cat.P1420). The concentration of the synthesized mRNA was measured using a NanoDrop ND-1000 spectrophotometer, and the 10-fold serially-diluted mRNA was used to generate the standard curve using the One Step PrimeScript™ RT-PCR Kit (Takara, Cat.064A). Briefly, viral RNA was extracted from plasma samples of HFRS patients and the real-time RT-PCR assays were conducted using the One Step Prime Script™ RT-PCR Kit.

### Measurement of antibodies and cytokines

Serum titres of IgM and IgG against HTNV nucleocapsid protein were measured using commercial ELISA kits (WANTAI BioPharm) following the manufacturer's instructions. Serum specimens were diluted in the ranges 1:10–1:640 for IgG and 1:5,120–327,680 for IgM. Optical density was measured at 450 nm (OD_450_) after addition of the blocking reagent. A multiplex-biometric immunoassay based on fluorescent microspheres conjugated with monoclonal antibodies specific for target cytokines was performed to assess serum cytokine levels (Bio-Rad, Cat.12007283). Concentration of 48-plex cytokines in serum samples was examined. The data were processed using the Luminex data collection software (version 6.1) [14,15].

### Virus-specific B-cell detection

PBMCs stored in liquid nitrogen were thawed, diluted using RPMI1640 supplemented with 10% heat-inactivated FBS (10^6^ cells/ mL), and stimulated with 2 µg/mL NP for three hours. Then, cells were cultured for an additional 16 h in the presence of BFA/Monensin Mixture (Multi Sciences, Cat.CS1002) at 37°C in 5% CO_2_ [16]. Subsequently, PBMCs were collected for staining for flow cytometric analysis as previously described [17]. Briefly, the PBMCs were stained with fluorescence labelled mAbs for 30 min on ice, including CD19-Percp-Cy5.5, CD21-PE-CF594, CD27-BV421, CD38-APC, CD80-PE, IgD-PE-Cy7, and dead cells were then excluded by fixable viability stain 700 (Cat.564997) for 15 min. After two washes, cells were resuspended in FACS buffer and immune phenotyping was carried out on a 12-laser Fortessa (BD Bioscience, USA) multiparameter flow cytometer. All antibodies and reagents used in the flow cytometry analysis were purchased from BD Biosciences, USA. Data were analysed using FlowJo version 10. The percentages of B-cell subsets were calculated and compared between groups or different time points.

### RNA sequencing and immune repertoire

RNA was purified from PBMCs using the RNeasy Plus Mini Kit (Qiagen, Cat.74134). RNA concentration and integrity were determined with the RNA Bioanalyzer (Agilent, USA). RNA samples were sequenced with 2 × 150 bp paired-end reads on the BGISEQ platform. Reads with low quality or adaptor contamination were filtered out, and the clean reads were aligned to the Genome Reference Consortium Human genome build 38 using HISAT2 [18]. In featureCounts, the Subread package was used to count reads aligned to each gene in order to quantify gene expression levels [19]. Differential gene expression analysis was carried out on the expression quantification results using the R package, limma-voom [20], in order to compare the differences between the severe and moderate groups, and between different time points. Gene set enrichment analysis (GSEA) was then applied for each comparison using the EGSEA R package. Blood transcription modules (BTMs) were employed in the gene set analysis. R version 3.6.1 was used in all the analyses.

For BCR sequencing, the complementary determining region 3 (CDR3) of BCR H chain was amplified in a multiplex PCR and sequenced with 2 × 150 bp paired-end reads on the Illumina xTen platform. Reads with low quality or adaptor contamination were filtered out. The filtered reads were aligned to BCR germline sequences using MiXCR [21]. VDJtools was used for downstream analysis including clonotype filtering, diversity analysis, and repertoire overlap.

### Statistical analysis

One-way analysis of variance (ANOVA) or Wilcoxon test was used in group comparisons. Pearson's correlation test was used to estimate the association between variables. Paired *t*-test was used for consecutive samples. We performed statistical analyses by using SAS version 9.4. Asterisks in each ﬁgure showed statistically significant differences (*P < 0.05; **P < 0.01).

## Results

### Characteristics of the study participants

**Figure 1.**
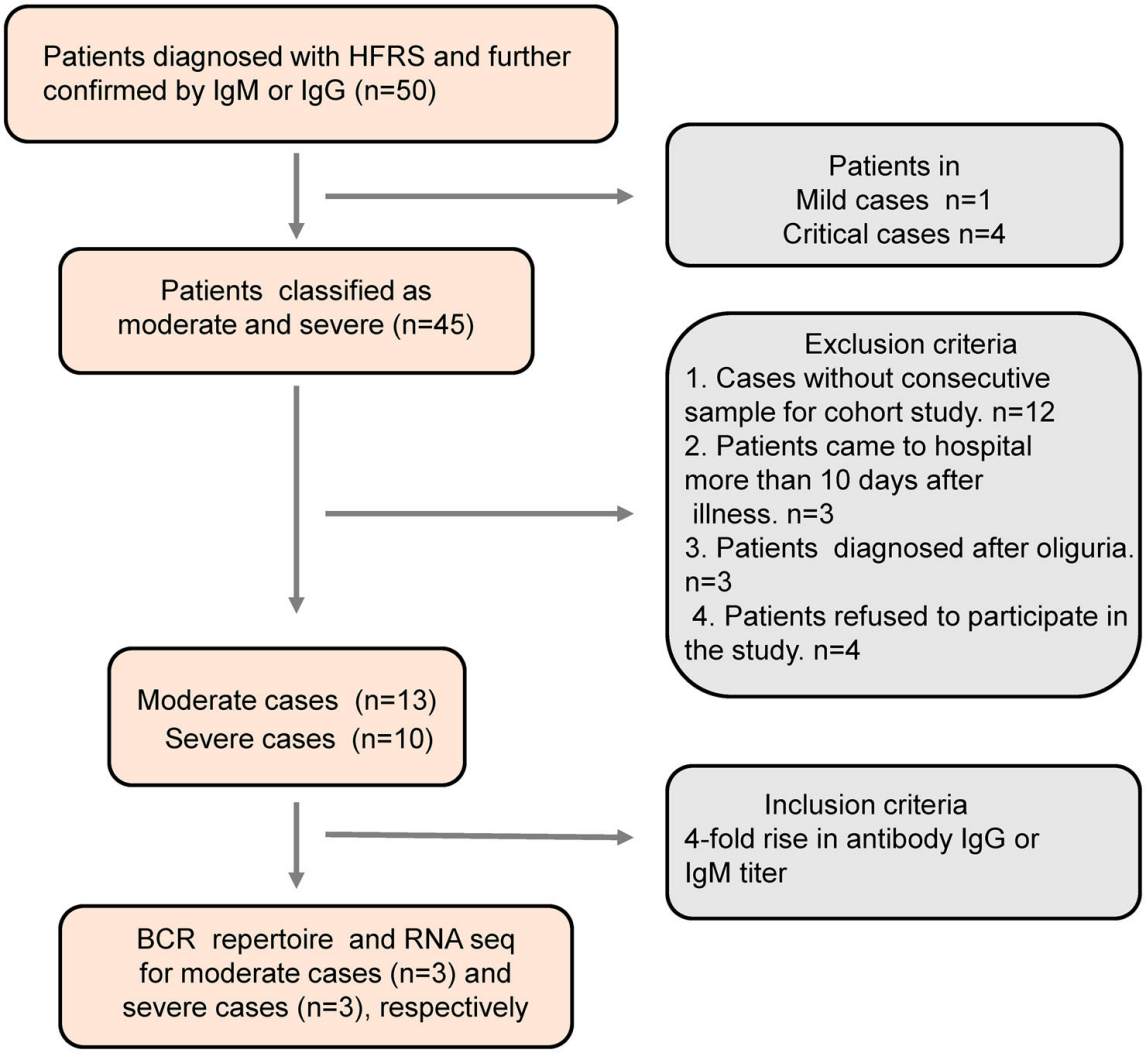
The flow chart of HFRS patient recruitment in Baoji Central Hospital in Shaanxi province from November 2018 to January 2019.

A total of 50 participants were enrolled from Baoji central hospital in western Shaanxi province from 28 November 2018 to 30 January 2019 during the HTNV peak season. Individuals were omitted from the study based on the following exclusion criteria: (1) cases without consecutive samples available for a longitudinal cohort study; (2) patients presenting to hospital >10 days after illness; (3) patients diagnosed after oliguria; (4) patients refusing to participate in the study. In total, 23 patients, comprising 13 moderate and 10 severe HFRS cases, were enrolled in this study (Figure 1). In addition, 10 age- and gender-matched healthy volunteers were also enrolled in Baoji central hospital as the control group from the department of physical examination during January 2019. Demographic and clinical features of the 23 test subjects are shown in Table 1. The median age of the moderate HFRS disease group was similar to that of the severe cases: 45 years (range 31–63) versus 49 years (range 30–60), whereas the hospitalization time differed significantly: 11 days (range 9–12) versus 17.5 days (range 17–22) (*P* < 0.001). For the 10 participants in the control group (male, 45.8%; median age, 48 years; interquartile range, 34–59 years), there were no significant differences in the demographic data compared with the 23 HFRS patients.

**Table 1.** Demographic and clinical signs of HFRS patients.

Characteristics	Moderate (*n* = 13)	Severe (*n* = 10)	*P-*value^a^
Demographic features	** **	** **	** **
Male	9 (69.2)	10 (100.0)	0.08
Age, median years (IQR)	45 (31–63)	49 (30–60)	0.86
Occupation	Farmer (9), others (4)	Farmer (5), others (5)	0.22
Days post symptom debut, median (IQR)	5 (4–6)	4.5 (4–6)	0.07
Days of hospitalization, median (IQR)	11 (9–12)	17.5 (17–20)	**<0**.**001**
Main lab finding on admission^b^			
APTT, median (IQR)	40.7 (38.4–43.9)	57.6 (53.8–62.3)	**<0**.**001**
D-dimer, median (IQR)	1.5 (0.8–2.1)	3.7 (2.4–5.1)	**0**.**004**
MCHC, median (IQR)	355 (350–357)	367 (363–369)	**0**.**043**
NEUT counts× 10^9^/L, median (IQR)	5.0 (3.9–5.5)	9.1 (4.3–16.9)	**0**.**046**
Platelet counts × 10^9^/L, median (IQR)	50.0 (33.0–65.0)	15.5 (7.0–21.0)	**0**.**002**
WBC counts × 10^9^/L, median (IQR)	12.5 (11.1–14.8)	20.4 (17.4–34.1)	**0**.**004**

Abbreviations: IQR, interquartile range; APTT, activated partial thromboplastin time; HGB, determination of haemoglobin in blood; MCHC, mean corpuscular haemoglobin concentration; NEUT, neutrophil; WBC, white blood cell.

^a^ Kruskal–Wallis test or t-test. Significant *P*-values are shown in bold.

^b^ APTT: normal range, 22–38; D-dimer: normal range, 0–1; MCHC: normal range, 316–354 g/L; NEUT counts: normal range, 1.8–6.3 × 10^9^/L; Platelet counts: normal range, 125–350 × 10^9^/L; WBCs: normal range, 3.5–9.5 × 10^9^/L. The normal ranges were referred to the National Health Commission of the People's Republic of China (WS/T 405-2012). Platelet counts and WBC counts were the maximum titres during hospitalization.

The HFRS patients presented 4–6 days after the development of symptoms for both moderate and severe groups. Levels of clotting factors, including activated partial thromboplastin time (APTT; *P* < 0.01) and D-dimer (*P *= 0.004), were significantly higher in the severe compared to the moderate group, suggesting that clotting enzyme activities were negatively affected by HTNV infection. Mean corpuscular haemoglobin concentrations (*P *= 0.043) and neutrophil counts (*P *= 0.046) were also relatively increased in the severe group. These data indicated severe blood vessel damage during acute viral infection (Table 1 and Supplementary Table 1).

### Effective NP-specific antibody responses associated with HTNV clearance

**Figure 2.**
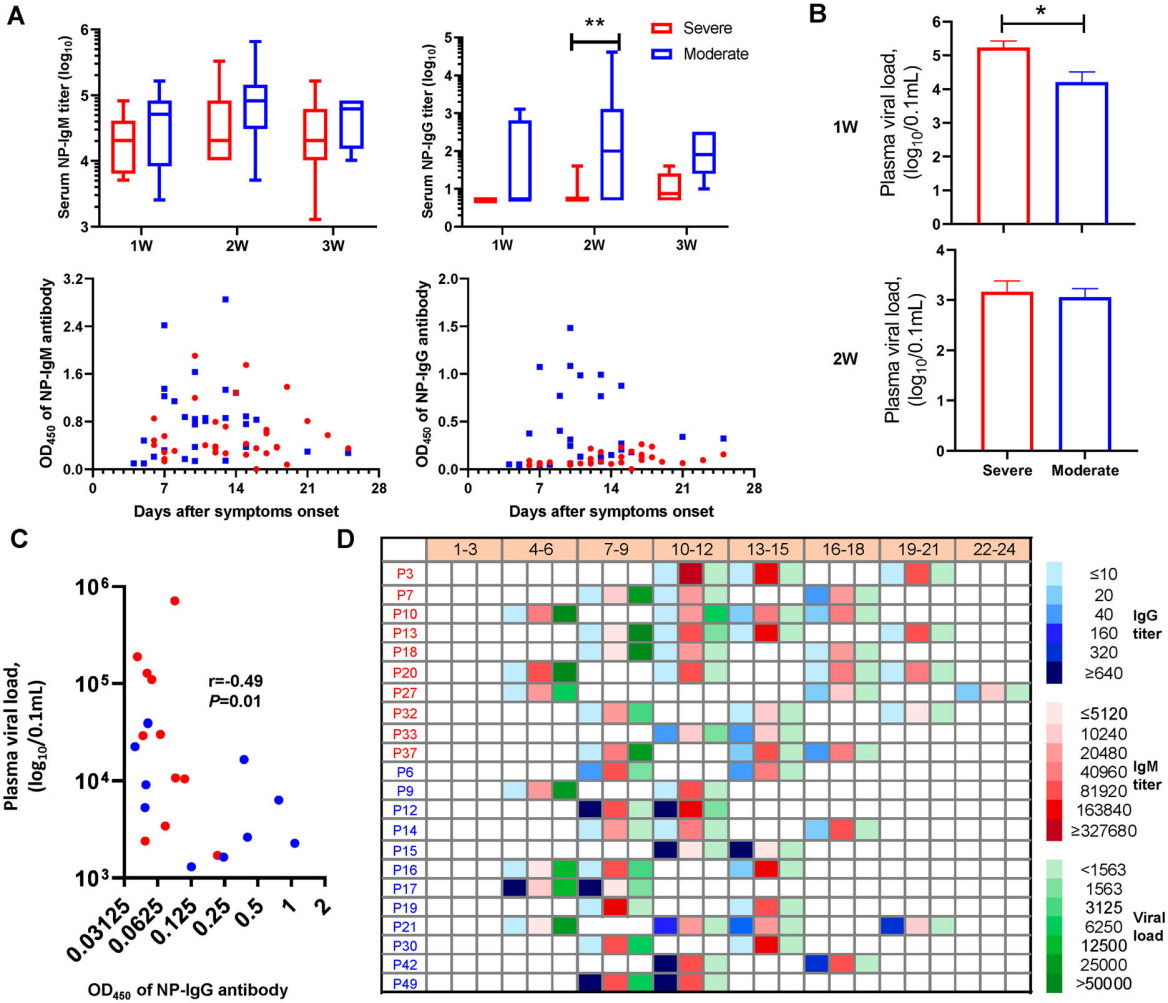
The antibody responses to the nucleocapsid protein among moderate and severe HTNV cases. (A) The IgM and IgG antibody titres against NP were examined by twofold dilutions and OD_450_ measurements in 1:10,240 and 1:40 diluted serum determined by a commercial ELISA kit in HTNV patients after symptom onset. Comparison of moderate and severe cases during acute phase was assessed by Kruskal–Wallis or Fisher's exact test. (B) Plasma viral load of patients at time different points was determined by real-time quantitative RT-PCR, and then compared to the first and second weeks by Kruskal–Wallis test. (C) Spearman's correlation coefficient was calculated between plasma viral load and NP-IgG titre using viremic samples. *X*-axis denotes the 1:40 diluted serum and reads the number at OD_450 nm_. *r* and *P* indicate the correlation coefficient and significance, respectively. (D) The dynamic changes of IgM and IgG to NP, and viral loads of moderate and severe cases at different time points. Data for P25 were not available. The blue, red, and green squares represent IgG, IgM, and virus copies/0.1 mL, respectively. In panels A–C and the first column of panel D, the blue and red colours represent the moderate and severe cases, respectively.

The sera from HTNV cases were analysed for the presence of NP-specific IgM and IgG antibodies. Only one case (10.0%) among the severe group was positive for IgG in the first two weeks, whereas seven (58.3%) in the moderate group were IgG positive (*P *= 0.03). Additionally, the reactivity level in the second week for IgG were more pronounced among the moderate than the severe group. Although the IgM antibody titres were positive for both moderate and severe groups, those of the moderate cases were slightly higher than the severe: 48,000 versus 29,257 in first week, and 111,736 versus 74,472 in the second week (OD_450_ values) (Figure 2A). These data suggested that the earlier and higher the NP-specific IgG and IgM appeared, the more likely HFRS developed mild disease.

Prior studies have suggested that the deficiency of virus-specific IgG was associated with persistent viremia [22,23]. Hence, we examined plasma viral loads in HFRS patients by quantitative RT-PCR. HTNV RNA was detectable in all severe patient samples collected one week after disease onset with a mean viral load in severe patients of 1.7 × 10^5^ copies/0.1 mL, compared to 1.6 × 10^4^ copies/0.1 mL in the moderate group (*P* < 0.05), which reached a comparable level in the second week (1.4 × 10^3^ vs. 1.1×10^3^ copies/0.1 mL), and cleared by the third week (Figure 2B). Further, NP-IgG titre had a strong inverse association (Spearman's *r* = −0.49, *P *= 0.01) with plasma viral load as demonstrated by the correlation analysis in HFRS cases (Figure 2C). Indeed, if the OD_450_ values of NP-specific IgM levels elevated to 0.8 at a 1:10,240 dilution or the NP-specific IgG increased to 0.2 at a 1:40 dilution post symptom onset, HFRS was more likely to be moderate (9/12) rather than severe (3/10). Importantly, all cases in the moderate group eventually reached the cut-off values, whereas only three cases in the severe group met this threshold (Figure 2D and Supplementary Table 2). Similar results were obtained for NP-specific IgG, where anti-HTNV IgG titre did not exceed 0.2 at a 1:40 dilution within 10 days of symptom onset in the severe group (Figure 2D); while higher anti-HTNV IgG titres (e.g. >0.2 at a dilution up to 1:40,960 dilution in P15) were observed in moderate cases as early as 14 days since symptom onset.

### Rapid responses of B-cell subsets to HTNV infection

**Figure 3.**
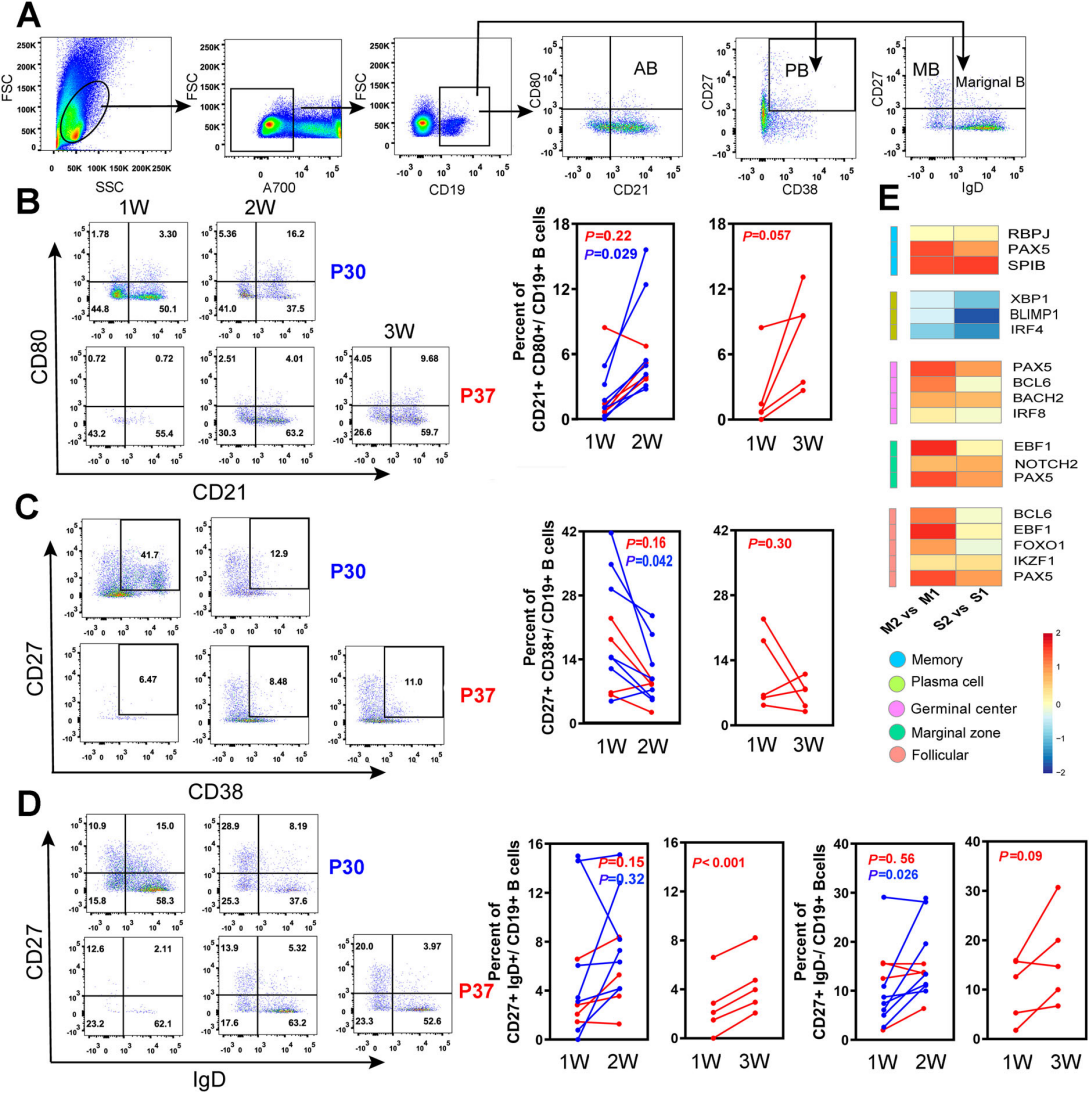
Dynamic analysis of B-cell subsets in HFRS patients. (A) FACS gating strategy for the measurement of functional B-cell subsets. Activated B cells (AB), marginal zone-like (marginal B), memory B cells (MB), and plasmablasts (PB), represented by CD21^+^CD80^+^, CD27^+^IgD^+^, CD27^+^IgD^−^, and CD27^+^CD38^+^ markers, respectively. (B–D) The changes of different B-cell subsets in both moderate and severe cases from first to third weeks after symptom onset in typical cases: the moderate case P30 and severe case P37. Paired *t*-test was used to assess the significance. The red line represents the severe group and the blue line represents moderate cases. (E) A heat map showing fold increase (red) or decrease (blue) in gene expression that is related to different B-cell subtypes. M2 and S2 represent the moderate and severe cases in week 2, respectively. Similarly, M1 and S1 represent the moderate and severe cases in week 1, respectively.

To ascertain the dynamics of functional B-cell subsets during acute HTNV infection, we measured the activated and reactive B cells in peripheral blood mononuclear cells (PBMCs) by flow cytometry. B-cell subsets were defined phenotypically as reported previously [22,24,25]: activated B cells (AB, CD19^+^CD21^+^CD80^+^), marginal zone-like B cells (marginal B, CD19^+^CD27^+^IgD^+^), memory B cells (MB, CD19^+^CD27^+^IgD^−^), and plasmablasts (PB, CD19^+^CD27^+^CD38^+^) (Figure 3A). In order to illustrate the dynamic trends of functional B cells, the flow plots of representative HTNV patients (moderate: P30, severe: P37) from the first to the third week after disease onset were plotted. We discovered that the AB subsets increased rapidly in the first two weeks, particularly in the moderate group, which was significantly higher than the severe group. The paired t-tests on the consecutive HTNV cases showed that the increase of HTNV-specific ABs (CD21^+^CD80^+^, *P *= 0.029) in the moderate group were significantly more rapid than severe cases, while a sharp decline in PB (CD27**^+^**CD38**^+^**, *P *= 0.042) occurred in the moderate group (Figure 3B and C). Similarly, MB subpopulations (CD27^+^IgD^−^) had a distinctive increase from week 1 to 2 (*P *= 0.026) in the moderate group; however, severe cases typically required approximately 3 weeks to generate diverse B-cell subsets: CD27^+^IgD^−^ (*P *= 0.09) and CD27^+^IgD^+^ (*P* < 0.001) (Figure 3D).

The fate of antigen-specific B cells is strictly regulated by a set of transcription factors [26]. The expression of IRF4, PRDM1 (encoding BLIMP1), and XBP1, which drive B cells into antibody-secreting cells, were lower in week 2 compared to week 1, particularly the PRDM1 transcript in the severe group. Other transcription factors, including Bach2, Notch2, IRF8, RBPJ, Pax5, Spi-B, and Bcl6, were increased in both severe and moderate groups at later time points, with a more pronounced rise in the moderate cases (Figure 3E). Although the expression of the transcription factors and gene regulatory network in B cells were not significantly different on the whole in the acute phase of infection, the moderate group had a more rapid reaction of activated B cells and antibody-secreting cells during the recovery process.

### The B-cell receptor repertoire diversity expansion during HFRS recovery

**Figure 4.**
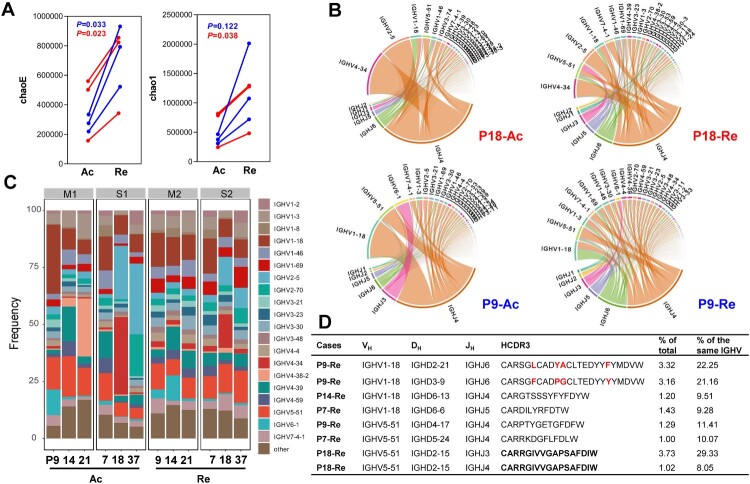
The increased BCR repertoire diversity in HFRS cases. (A) BCR diversity, assessed by chaoE and chao1, was rapidly expanded in cases with antibody titres producing fourfold increase in either IgM or IgG subtype. Ac represents the acute phase and Re represents the recovery phase before and after fourfold rises in antibody titres. (B) V and J gene rearrangement characteristics from two typical cases, moderate P9 and severe P18, were depicted based on the read number of genes. The top 15V genes and all of the J genes were labelled in the circos plots. Genes from the same major class were assigned the same color. For example, IGHV4-34 and IGHV4-39 are shown in pink, and IGHV2-5 and IGHV2-70 are shown in orange. (C) IGHV segment usage ratio in HTNV patients. Packaged bars indicate the respective percentages of top 20 V genotypes. (D) Dominant antibody sequence characteristics identified following a fourfold rise in titre. The proportion of IGHV1-18 and IGHV 5-51 were calculated for the total and corresponding IGHV genotype. The region of V_H_, D_H_, and J_H_ was confirmed by IgBlast in NCBI.

The diversity of the B-cell receptor **(**BCR) repertoire is the result of the somatic recombination process, which brings together one each of the variable (V), diversity (D), and joining (J) segments to form the exon of an immunoglobulin heavy chain, with one each of the V and J segments forming a light chain [27]. The CDR3 sequence has been previously used to represent BCR repertoires after infection or vaccination [28]. Herein, 12 PBMC samples from the HFRS patients, with at least a fourfold rise (before fourfold rise, acute phase: Ac; after fourfold rise, recovery phase: Re) in antibody titres, were employed to assess dynamic BCR changes by using a variety of diversity indices, such as chaoE, Chao 1 and the Shannon–Wiener index. According to the paired analysis, diversity was significantly increased in HFRS patients in the recovery phase for both chaoE (276,687–748,946 in the moderate group, *P *= 0.03; 418,403–694,136 in the severe group, *P *= 0.023) and Chao1 indexes (384,744–1,268,727 in the moderate group, *P *= 0.12; 619,647–1,023,848 in the severe group, *P *= 0.038) (Figure 4A and Supplementary Table S3). The diversity index Chao1 increased 2.6-fold in the moderate group, whereas the severe group had a less pronounced 1.7-fold rise (*P* = 0.049), with similar results also found for the chaoE index. For example, the top 10 proportions of VJ composition in P9 (a moderate case) and P18 (a severe case) in the acute phase were 67.9 and 79.7, while they were 41.8 and 54.1 after a fourfold antibody titre rise (Figure 4B and Supplementary Figure 1). Notably, the moderate cases typically required an average of 11.3 days to achieve a fourfold rise in antibody titre, while the severe cases required 15.7 days (Supplementary Table S3). These data suggested the BCR diversity of the moderate group may expand more quickly and require a shorter time to obtain a high antibody level during the recovery phase.

Unexpectedly, one or two V genes were dominant in the acute infection period, and the proportions of them gradually declined during the recovery period. For example, the proportion of IGHVP4-38-2 in the acute phase of patient 21 was 25.4%, whereas it sharply declined to 0.007% in the recovery phase (Figure 4C). Interestingly, irrespective of the prominent V gene types during the acute phase, the IGHV1-18 and IGHV5-51 types always predominated in the recovery phase, with the changes ranging from 7.9% to 15.4% and 7.1% to 14.1%, respectively (Figure 4C). Additionally, after a fourfold rise in antibody titre, both of the top two CDR3s derived from patient P9 belonged to IGHV1-18 and accounted for 43.4% of total IGHV1-18, with only four amino acid differences between them (Figure 4D). Similarly, a CDR3 in P18 composed of different J genes (IGHJ3 and IGHJ4) accounted for 37.4% of total IGHV5-51. Moreover, the J gene only had six genotypes and the J4 clone consistently remained dominant ranging from 41.6% to 81.8% during the recovery period (see Supplementary Figure 2). In both moderate and severe cases, the combinations of IGHJ4 and IGHV1-18/IGHV5-51 became the predominant paired types.

Combined with the analysis of NP-specific antibodies in Figure 2D, we explored further the diversity changes in severe cases, irrespective of whether the titre level reached the cut-off values during the recovery phase. For example, before the fourfold increase in antibody titre for cases P7, P18 and P37, the ChaoE indexes were 162,137, 577,193 and 516,775, respectively. Yet, after a fourfold increase, the diversity ChaoE index in P18 was 353,966, while P7 and P37 were 880,298 and 848,144, respectively. Therefore, although the antibody titre did not reach the cut-off value for P18, the diversity index also increased, despite always being lower than the other two severe cases who ultimately met the threshold (see Supplementary Table 3). This suggests that the diversity of the immune repertoire is closely related to the production of specific antibodies.

### Overexpression of proinflammatory genes in severe cases

To further investigate the immune responses during acute HTNV infection, we compared the expression profile between moderate and severe cases, as well as before and after fourfold rise in antibody titres. Differential gene expression analysis revealed only two genes between moderate and severe cases at both time points: sialic acid binding Ig like lectin 14 (SIGLEC14, adjusted *P*: 11 × 10^−4^ and 5.7 × 10^−4^) and an unannotated transcript ENSG00000273837 (adjusted *P*: 1.6 × 10^−5^ and 1.8 × 10^−5^) that were the most significantly upregulated in severe but not in moderate cases (S-Ac vs. M-Ac and S-Re vs. M-Re). It is noteworthy that SIGLEC14 regulates conversion of plasminogen (PLG) to plasmin, which subsequently degrades fibrin into soluble forms, including D-dimer, an important clinical biomarker of fibrinolytic activity. This was consistent with the clinical diagnosis of an unusual increase of D-dimer in severe HFRS cases (Table 1). We also analysed differences between time points among moderate and severe cases (see Supplementary Table 4). We discovered only two genes differentially expressed over time in moderate cases (M-Re vs. M-Ac), while 307 were found in severe cases (S-Re vs. S-Ac) that mainly functioned in immune responses (e.g. CCR4, CCR10 and IL1R2), cytokine production (e.g. CD4, FOXP3 and S1PR3), and the adaptive immune response (e.g. CD1E, CD224, PRDM1, CTLA4 and JAM3). Notably, the MYCL proto-oncogene, a bHLH transcription factor that plays an important role in the maintenance of cell viability, was significantly over-expressed in both groups in the recovery phase (adjusted *P*: 0.03 and 0.006).

**Figure 5.**
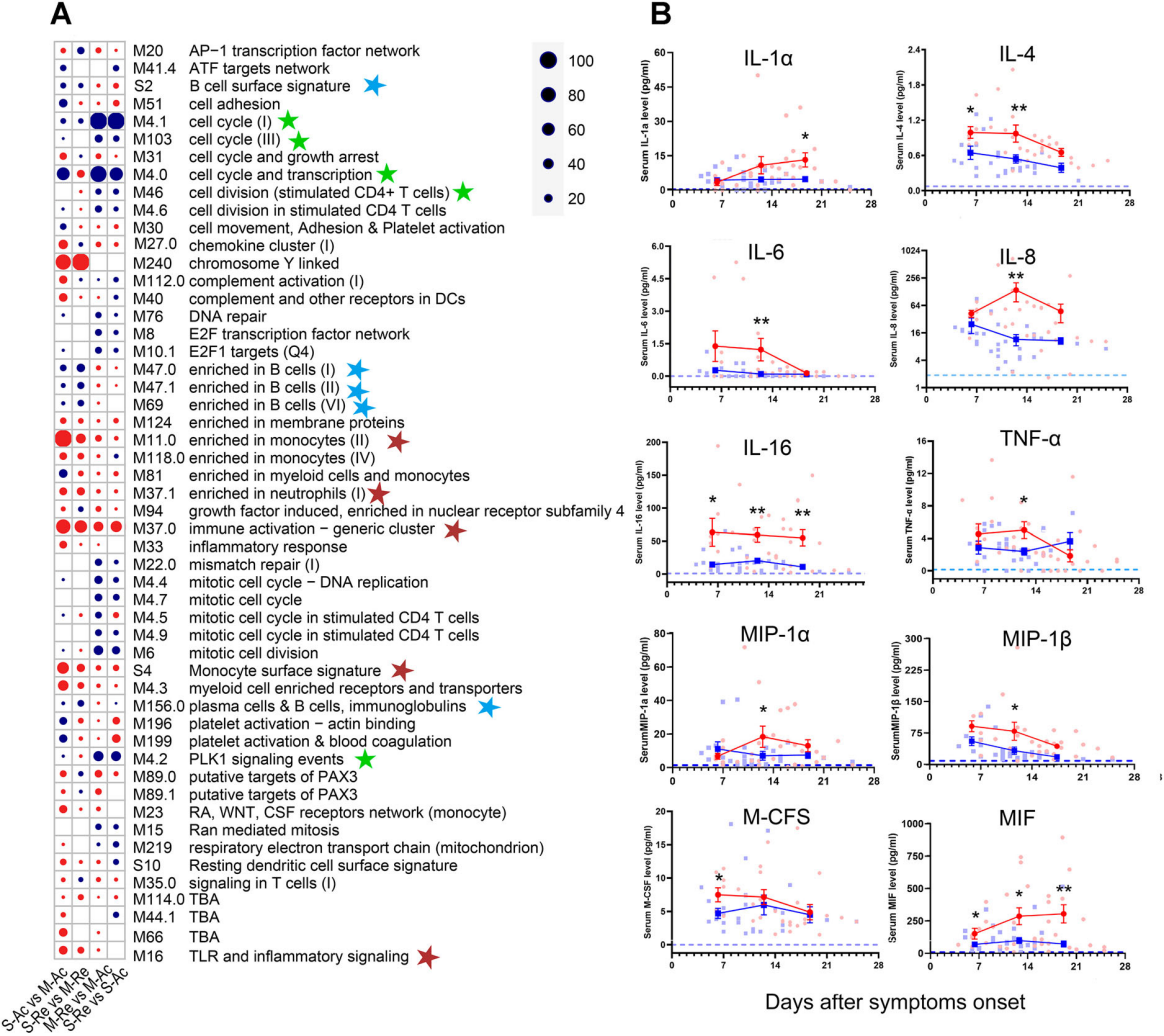
Gene expression array analysis and related serum cytokine in HTNV patients. (A) The gene set analysis shows the identifiers and names of the top 20 BTM gene sets of each comparison sorted by the significance score. Red indicates positive enrichment of a gene set and blue indicates negative enrichment. Circle size is proportional to the significance score that assigns high scores to the gene sets with strong fold-changes and high statistical significance. The blue, brown and green stars represent gene sets enriched in B cells, monocytes and neutrophils, and cell cycle, respectively. S-Ac: severe cases in the acute phase; M-Ac: moderate cases in the acute phase; S-Re: severe cases in the recovery phase; M-Re: moderate cases in the recovery phase. (B) Dynamic comparison of serum cytokine concentrations in both moderate and severe groups during the 3-week period post symptom onset. Blue squares, red dots and blue dashed lines represent means of moderate, severe and health group, respectively. The mean +/- standard error is shown for each group and the *P*-value is determined by *t*-test or Kruskal–Wallis test. Statistical significance was represented by asterisk, * *P *< 0.05, ** *P *< 0.01.

In addition to differentially expressed genes between groups and over time, we analysed several pre-defined functionally related gene sets (i.e. blood transcription modules, BTM) (see Supplementary Table 5). Figure 5A presents the top 20 gene sets for each comparison. Positive enrichment was found in the gene sets related to cytokines and chemokines between different time points, consistent with the aforementioned results (see Supplementary Table 4). First, the gene expression in immune activation (M37.0) and monocyte (M11.0 and S4) increased (with a significance score >50) in the severe group, while the B-cell-related gene transcripts (S2, M47.0, M47.1, M69, and M156.0) were negatively enriched in the severe group at both time points (S-Ac vs. M-Ac and S-Re vs. M-Re) (Figure 5A). Second, the proinflammatory gene sets (M11.0, M37.0, M37.1, and S4) were upregulated in both groups at a later time point. Similarly, genes enriched in B cells or on their surfaces also increased over time in both groups, however, the cell cycle, transcription and division gene sets (M4.0, M4.1, M4.2, M46, M103), significantly declined over time (M-Re vs. M-Ac and S-Re vs. S-Ac).

The dynamics of B-cell subsets from activation to functional responses are closely regulated by numerous cytokines [7], revealed by transcriptome analysis. In order to further verify these results, cytokines in the sera of HTNV patients were measured using a high-sensitivity multiplex ELISA [29]. As expected, elevated concentrations of proinflammatory cytokines were observed during the acute phase of infection in the severe group, including IL-1α, IL-6, IL-8, IL-16, and TNF-α. These cytokines decreased slowly with the exception of IL-1α (Figure 5B). In addition, chemokines, macrophage inflammatory protein-1α (MIP-1α), MIP-1β, and macrophage colony stimulating factor (M-CSF) were also more highly expressed in the severe group. The concentrations of anti-inflammatory cytokines, IL-4 and macrophage migration inhibitor factor (MIF), were significantly higher in the severe group from the first week post symptom onset, which was consistent with the transcription analysis (Figure 5B).

## Discussion

The humoral immune response plays an important role in controlling infections and vaccine responses following immunization [30]. This has been reported in many viral diseases, such as severe fever with thrombocytopenia syndrome, seasonal influenza and Ebola haemorrhagic fever [22,30,31]. However, the role of humoral immunity in the spectrum of clinical presentation with HFRS has not been elaborated clearly to date. Herein, we constructed a cohort study to explore the mechanism of B cells in the immune defence against HTNV infection.

One of the most intriguing findings from the present study is that there was a more rapid and robust proliferation of HTNV-specific activated memory B cells from the first to the second week post symptom onset in the moderate compared to the severe group. Accordingly, the response of antibody-secreting plasma cells peaked in the first week, similar to previous studies with B-cell subsets increasing after infection or vaccination [30]. The speed of activation and memory B-cell changes were also significantly more rapid in the moderate patients. Evidently, the rapid expansion of HTNV-specific B cells during the early stages of infection is closely associated with disease outcome.

The fate of HTNV-activated B cells is strictly controlled by the expression levels of transcription factors. Previous studies have reported that the expression of BLIMP1, IRF4, and XBP1 genes together regulated plasma cell differentiation and antibody production [31,32]. Herein, we observed a decline of these genes at the transcription level in both groups after a fourfold rise in antibody titres, which is consistent with decreases in the CD27^+^CD38^+^ plasma cells. However, a set of transcription factors that help B cells maintain their identity, such as Pax5, IRF8, and Bcl6, were upregulated during the recovery phase, particularly in the moderate group, suggesting that B cells gradually returned to normal status.

In this study, we found relatively conserved antibody recombinants in HFRS cases, such as IGHV1-18 and IGHV5-51. Interestingly, Jiao and colleagues reported that the V genes in six strains of monoclonal antibody (MAb 4-5, A5, A9, A11, B2, and D6) were IGHV5-51, 1-46, 1-8, 1-8, 1-69 and 1-6. In particular, MAb 4–5 possessed neutralizing activity against severe fever with thrombocytopenia syndrome virus [33,34], which suggests that antibodies possessing the V gene of IGHV5-51 might have neutralizing activity against HTNV infection. Additionally, the germ line analysis of scFv A5 revealed it to be the IGHV4-4 gene [35], which ranked in the top 20 V genes in our study and even accounted for 0.3% in case P9. However, the neutralizing efficacy of IGHV5-51 and IGHV4-4 against HTNV requires future study.

It has been reported that upregulation of cytokine levels occurs in HTNV patients and is closely related to disease severity [15,36–38]. Similarly, cytokines affecting B cells, such as IL-4 which promotes IgG1 production, were significantly higher in the severe cases with lower antibody titres. We hypothesize that cytokine overexpression in the early phase of HTNV infection may lead to a hypercytokinemia impairing the viral elimination and exacerbating pathological manifestations. In agreement with previous studies, the dysregulation observed in the severe group during the acute phase of the infection mainly comprised the upregulation of secreted inflammatory mediators, such as IL-8, MIP-1α, MIP-1β, M-CFS, MIF, and TNF-α [39,40]. Of particular note, the proinflammatory cytokine IL-8, has a role in aggravating vascular permeability [41]. Besides, IL-8 and monocyte chemoattractant protein-1 (MCP-1) are associated with thrombocytopenia and may contribute to platelet activation, either by their chemoattractant properties or by their effects on endothelial cell permeability [42]. Indeed, MIP-1β and MCP-1 produced by monocytes, dendritic cells, and NK cells recruit immune cells to sites of inflammation [42,43]. These findings are consistent with clinical presentation of HFRS patients, including hyperaemia, oedema, and thrombocytopenia. Therefore, the precise mechanisms whereby cytokines affect B-cell maturation and serve to prevent HTNV infection warrants further studies to potentially improve triaging of patients and the development of efficacious therapeutics and vaccines.

In summary, we have investigated the humoral immune responses during HTNV infection by characterizing the dynamic variation of B-cell subsets, the BCR immune repertoire and cytokine profiles during the viral infection process. We provide evidence that in HFRS patients with B cells that possess a prompt activation rate, high clonal diversity, and low cytokine storm damage, they are typified by an increased ability to produce virus-specific antibodies and that eventually, this disease is more likely to develop a mild illness and a rapid recovery. These results establish the association between humoral immune response (particularly HTNV-specific antibodies) and the disease severity of HFRS cases that could potentially be used to predict and improve clinical outcomes of HTNV infection. More importantly, we have also identified several novel candidate peptides with potential neutralizing activity against HTNV, which warrants investigation in future studies.

## Author contributions

Weifeng Shi and Weijia Xing designed and supervised this study. Yaoni Li collected the samples and Chuansong Quan, Jiming Gao, Zhenjie Zhang, Xiaolin Jiang, Chuanmin Ma and Qian He performed the experiments. Chuansong Quan, Peihan Wang, Weijia Xing, and Lei Gao performed statistical analysis. Hua Tang provided technical assistance. Chuansong Quan and Peihan Wang wrote the manuscript. Weifeng Shi, Michael J. Carr, and Yuhai Bi edited the paper.

## Supplementary Material

Supplemental Material

Supplementary__Fig1-2_and_Table1-5_clean.docx

## Data Availability

All data used in this study, including raw scRNA-seq and scBCR-seq data, filtered expression matrix and BCR heavy chain CDR3 annotations were submitted to GEO under the accession number GSE158712.
