# Supplementary material for: Rapid humoral immune responses are required for recovery from haemorrhagic fever with renal syndrome patients
Source: Emerg Microbes Infect. 2020 Oct 21;9(1):2303–14. doi: 10.1080/22221751.2020.1830717 (PMC8284976; doi:10.1080/22221751.2020.1830717)
Supplement: Supplementary__Fig1-2_and_Table1-5_clean.docx [file TEMI_A_1830717_SM0883.docx]

**Supplementary materials**

**
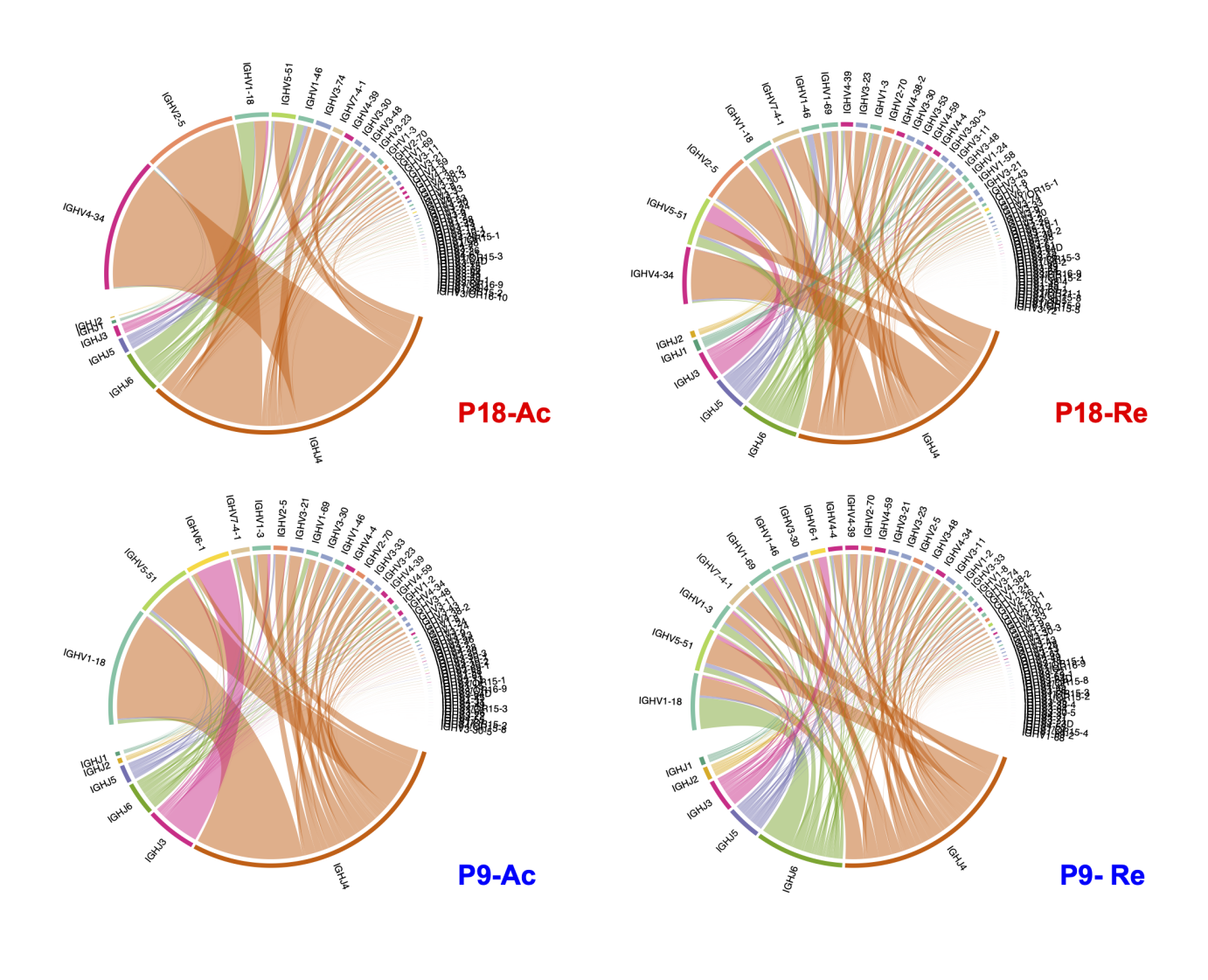
**

**Supplementary Figure 1. Rearrangement of IGHV and IGHJ genes in HTNV patients.**

V and J gene rearrangement characteristics from two typical cases, moderate P9 and severe P18, were depicted based on the read number of genes. “Ac” and “Re” represent cases in the acute and recovery phase, respectively

**
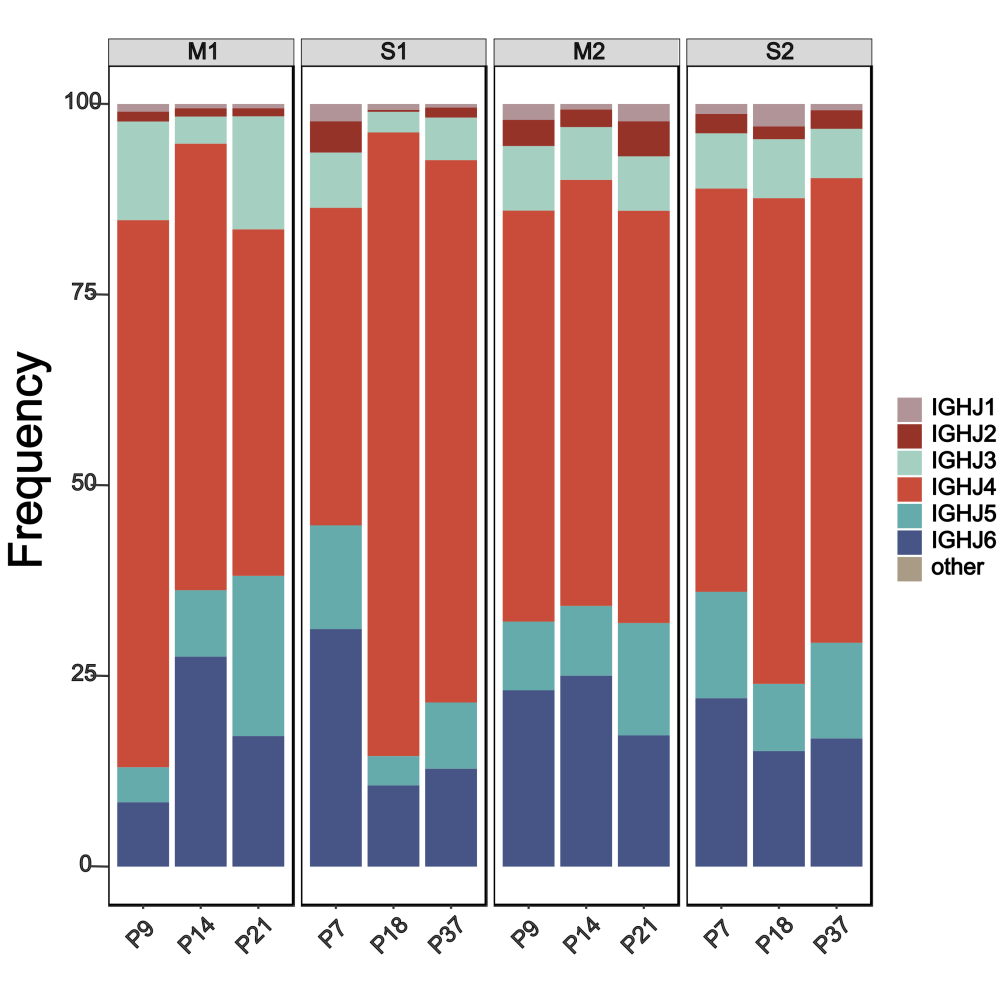
**

**Supplementary Figure 2. IGHJ segment usage ratio in HTNV patients.**

**Supplementary Table 1. Characteristics of patients with hemorrhagic fever with renal syndrome.**

| **Patients** | **TM** | **Creatine**  **Level ^a^, µmol/L** | **Blood urea level, mmol/L** | **Hyperemia**  **and edema** | **The lowest volume of**  **continuous 5d urea, mL** | | | | | **Platelet level,**  **10**^3^**/µL** | **WBC level,**  **10**^9^**/L** |
| --- | --- | --- | --- | --- | --- | --- | --- | --- | --- | --- | --- |
|  |  |  |  |  | 1d | 2d | 3d | 4d | 5d |  |  |
| **Severe** | | | | | | | | | | | |
| **P3** | 39.6 | 496 | 19.8 | ++ | 900 | 1580 | 1150 | 790 | 3330 | 17 | 15 |
| **P7** | 41 | 772 | 43.4 | +++ | 220 | 230 | 240 | 420 | 2155 | 9 | 20.9 |
| **P10** | 40 | 852 | 33.5 | +++ | 503 | 180 | 139 | 97 | 215 | 33 | 30.7 |
| **P13** | 40 | 952 | 43.3 | +++ | <500 | 680 | 800 | NA | 1140 | 14 | 42.3 |
| **P18** | 39.5 | 888 | 32.5 | +++ | 140 | 85 | 76 | 54 | 175 | 21 | 18.6 |
| **P20** | 40 | 1041 | 26.9 | ++ | 570 | 130 | 133 | 255 | 450 | 54 | 17.4 |
| **P27** | 39.8 | 469 | 30.2 | +++ | 505 | 290 | 575 | 690 | 945 | 6 | 35.2 |
| **P32** | 39.6 | 775 | 39.7 | ++ | 45 | 310 | 370 | 597 | 590 | 6 | 34.1 |
| **P33** | 37.6 | 790 | 37.2 | +++ | NA | 300 | 500 | 1400 | NA | 21 | 17.2 |
| **P37** | 40 | 760 | 25.7 | +++ | 1200 | 800 | 600 | 780 | 650 | 7 | 19.8 |
| **Moderate** | | | | | | | | | | | |
| **P6** | 38.5 | 379 | 28.4 | - | 150 | 2730 | 1430 | 3310 | 5280 | 52 | 14.8 |
| **P9** | 39.8 | 124 | 7.4 | + | 200 | 1850 | 3020 | NA | NA | 65 | 19.4 |
| **P12** | 39.1 | 300 | 12.7 | + | 540 | 1015 | 2329 | 3700 | 3750 | 49 | 11.9 |
| **P14** | 39.2 | 151 | 11 | +++ | >500 | 2850 | 2620 | NA | NA | 57 | 11.1 |
| **P15** | 38.3 | 183 | 14.5 | - | 850 | 1400 | 2100 | 3150 | 1800 | 50 | 11.9 |
| **P16** | 40 | 130 | 5.9 | - | >400 | 2220 | 1170 | 965 | 1170 | 29 | 12.5 |
| **P17** | 39 | 129 | 10.3 | - | >50 | 1130 | 470 | 480 | 900 | 46 | 5.7 |
| **P19** | 39.5 | 311 | 13.2 | - | 1800 | 2650 | 5740 | NA | NA | 112 | 8.8 |
| **P21** | 39.3 | 332 | 31.2 | + | 1920 | 3750 | 6150 | 4050 | 4140 | 21 | 13.7 |
| **P25** | 38.9 | 324 | 22.3 | + | 1200 | 1635 | 1255 | 1680 | 3125 | 16 | 22.8 |
| **P30** | 38.8 | 156 | 9.1 | - | NA | NA | 5050 | 6000 | NA | 130 | 10.4 |
| **P42** | 39 | 212 | 14.6 | - | 700 | 1640 | 1970 | 3000 | 3880 | 33 | 17.3 |
| **P49** | 38.5 | 110 | 5.8 | + | NA | NA | NA | NA | NA | 185 | 14.8 |

Abbreviation: TM, Temperature; NA, not available; WBC, White blood cell.

^a^ Except for volume of urea and platelet levels, other parameters were selected based on the highest levels during hospitalization.

**Supplementary Table 2. The antibody titers and viral loads of hemorrhagic fever with renal syndrome patients at different time points.**

| **^a^ Patients** | **^b^ IgG titer** | **IgM titer** | **Viral**  **loads** | **Patients** | **IgG titer** | **IgM titer** | **Virus**  **loads** |
| --- | --- | --- | --- | --- | --- | --- | --- |
| P3-10 | 0.066 | 1.906 | 0 | P6-10 | 0.246 | 0.841 | 1634 |
| P3-15 | 0.07 | 1.751 | 0 | P6-15 | 0.207 | 0.757 | 0 |
| P3-19 | 0.078 | 1.384 | 0 | P9-5 | 0.051 | 0.482 | 38774.5 |
| P7-1 | 0.066 | 0.287 | 30094 | P9-11 | 0.132 | 0.859 | 0 |
| P7-7 | 0.073 | 0.354 | 0 | P12-7 | 1.074 | 1.226 | 2272.5 |
| P7-16 | 0.186 | 0.354 | 0 | P12-10 | 1.085 | 1.637 | 0 |
| P7-18 | 0.237 | 0.378 | 0 | P14-7 | 0.04 | 0.316 | 0 |
| P10-6 | 0.089 | 0.485 | 714000 | P14-10 | 0.059 | 0.751 | 0 |
| P10-12 | 0.109 | 0.382 | 10514. 7 | P14-16 | 0.179 | 0.831 | 0 |
| P10-15 | 0.13 | 0.427 | 0 | P15-9 | 1.483 | 0.136 | 0 |
| P10-17 | 0.139 | 0.625 | 0 | P15-12 | 0.99 | 0.144 | 0 |
| P13-7 | 0.055 | 0.172 | 110342 | P16-5 | 0.039 | 0.098 | 22424.7 |
| P13-10 | 0.048 | 1.198 | 2400 | P16-8 | 0.048 | 1.144 | 5304.7 |
| P13-14 | 0.056 | 1.288 | 0 | P16-14 | 0.15 | 1.285 | 0 |
| P13-21 | 0.064 | 0.808 | 0 | P17-6 | 0.375 | 0.213 | 16514 |
| P18-7 | 0.05 | 0.133 | 128716.3 | P17-9 | 0.402 | 0.169 | 2621 |
| P18-11 | 0.061 | 0.403 | 0 | P19-7 | 0.05 | 2.417 | 0 |
| P18-18 | 0.125 | 0.365 | 0 | P19-13 | 0.083 | 2.856 | 0 |
| P20-6 | 0.041 | 0.853 | 189666.7 | P21-4 | 0.051 | 0.098 | 39479.7 |
| P20-12 | 0.059 | 0.796 | 0 | P21-10 | 0.311 | 0.375 | 0 |
| P20-17 | 0.095 | 0.601 | 0 | P21-15 | 0.27 | 0.377 | 0 |
| P20-23 | 0.095 | 0.573 | 0 | P21-21 | 0.34 | 0.298 | 0 |
| P27-6 | 0.09 | 0.406 | 10729.3 | P21-25 | 0.324 | 0.277 | 0 |
| P27-17 | 0.152 | 0.268 | 0 | P30-7 | 0.049 | 1.352 | 9073.3 |
| P27-25 | 0.155 | 0.35 | 0 | P30-13 | 0.126 | 1.332 | 1301 |
| P32-8 | 0.073 | 0.307 | 3443 | P42-11 | 0.984 | 0.809 | 0 |
| P32-13 | 0.079 | 0.269 | 0 | P42-15 | 0.878 | 0.887 | 0 |
| P32-19 | 0.074 | 0.08 | 0 | P49-9 | 0.769 | 0.875 | 6315.7 |
| P33-12 | 0.216 | 0.28 | 1717.7 | P49-12 | 0.766 | 0.864 | 0 |
| P33-15 | 0.231 | 0.244 | 0 |  |  |  |  |
| P37-7 | 0.046 | 0.557 | 29300 |  |  |  |  |
| P37-13 | 0.181 | 0.715 | 0 |  |  |  |  |
| P37-18 | 0.262 | 0.664 | 0 |  |  |  |  |

^a^ The patient numbers were expressed as “x-y”, “x” meaning patient number, “y” meaning patient collection time after onset of syndrome.

^b^ In order to easily observe differences between groups, titers that exceeded 0.2 in the IgG group are highlighted in purple, as well as 0.8 in the IgM group highlighted in yellow.

**Supplementary Table 3. Clone diversity index from selected hemorrhagic fever with renal syndrome patient samples.**

| Patients | Clinic type | IgG titer | IgM titer | Reads | Clone | ChaoE | Chao1 | Shannon  Wiener Index | Days after syndrome onset |
| --- | --- | --- | --- | --- | --- | --- | --- | --- | --- |
| P7-Ac | Severe | 0 | 10240 | 7050259 | 441526 | 577193 | 822161 | 8.176 | 7 |
| P7-Re | Severe | 40 | 20480 | 7598814 | 699556 | 880298 | 1301794 | 10.0252 | 18 |
| P18-Ac | Severe | 0 | 5120 | 5863015 | 111168 | 162137 | 245289 | 4.546 | 7 |
| P18-Re | Severe | 0 | 20480 | 7340074 | 280469 | 353966 | 485692 | 7.1499 | 11 |
| P37-Ac | Severe | 0 | 40960 | 11597650 | 516775 | 516775 | 791492 | 6.9357 | 7 |
| P37-Re | Severe | 40 | 61440 | 8275988 | 701103 | 848144 | 1284058 | 9.5752 | 17 |
| P9-Ac | Moderate | 0 | 20480 | 6511760 | 205803 | 275244 | 377044 | 5.9665 | 4 |
| P9-Re | Moderate | 0 | 81920 | 6542300 | 596596 | 793003 | 1073179 | 9.6191 | 10 |
| P14-Ac | Moderate | 0 | 20480 | 7467664 | 175181 | 220140 | 310185 | 6.2811 | 6 |
| P14-Re | Moderate | 10 | 81920 | 6617896 | 391545 | 522340 | 720833 | 8.315 | 15 |
| P21-Ac | Moderate | 0 | 2560 | 6152604 | 241263 | 334678 | 467002 | 5.9956 | 3 |
| P21-Re | Moderate | 160 | 20480 | 5903142 | 671307 | 931496 | 1248921 | 10.4174 | 9 |

**Supplementary Table 4. Differentially expressed genes detected between moderate and severe cases, as well as before and after 4-fold rise in antibody titers.**

**Supplementary Table 5. Enriched BTM gene sets identified using EGSEA that combines results from multiple algorithms.**

The supplementary tables 4 and 5 are in the xlsx format, and they are too large to be merged into the single PDF. We are pleased to share these files by other methods.
